# Pneumococcal Carriage in the Sahel Region of Burkina Faso before a 13-Valent Pneumococcal Conjugate Vaccination Campaign

**DOI:** 10.4269/ajtmh.24-0746

**Published:** 2025-04-22

**Authors:** Robert Lamoussa Zoma, Lana Childs, Issa Ouedraogo, Guetwendé Sawadogo, T. Félix Tarbangdo, Aristide Zoma, Soufiane Sanou, Brice Bicaba, Simon Sanou, Fahmina Akhter, Mahamoudou Ouattara, Jennifer R. Verani, Lesley McGee, Miwako Kobayashi, H. Flavien Aké

**Affiliations:** ^1^ Davycas International, Ouagadougou, Burkina Faso;; ^2^ Infectious Disease Programs, CDC Foundation, Atlanta, Georgia;; ^3^ Direction de la prévention par la vaccination, Ministère de la Santé et de l’Hygiène Publique, Ouagadougou, Burkina Faso;; ^4^ Unité de Bactériologie, Centre Muraz, Bobo-Dioulasso, Burkina Faso;; ^5^ Institut National de Santé Publique, Centre des Opérations de Réponse aux Urgences Sanitaires, Ouagadougou, Burkina Faso;; ^6^ Division of Bacterial Diseases, Centers for Disease Control and Prevention, Atlanta, Georgia

## Abstract

Burkina Faso introduced 13-valent pneumococcal conjugate vaccine (PCV13) in 2013 and achieved >90% three-dose coverage. Recently, the Sahel Region has experienced a security crisis, resulting in decreasing PCV13 coverage. We examined pneumococcal carriage before a mass PCV13 campaign in the Sahel Region in 2022. In January and February 2022, we conducted a cross-sectional, age-stratified pneumococcal carriage study among healthy individuals in Dori, the capital of the Sahel Region. We collected nasopharyngeal (all participants) and oropharyngeal swabs (participants 5 years old and older). Pneumococci isolated by culture were serotyped by polymerase chain reaction and/or Quellung. We evaluated overall and vaccine serotype pneumococcal carriage prevalence by age group. Among 1,079 participants, overall pneumococcal carriage prevalence was 57.2%; carriage was highest in children 1 year old (71.8%) and 1–11 months old (69.7%) and lowest in participants 15 years old or older (30.0%). Vaccine serotype carriage prevalence was 12.8%, ranging from 5.6% in participants 15 years old or older to 17.8% in children 5–14 years old. PCV13 vaccination history was unknown for 59.6% of age-eligible children. Among children with card-confirmed or verbally reported PCV13 history, most (99.0%) had no history of PCV13 receipt. Eight years after PCV13 introduction and in a conflict-affected area with declining PCV13 coverage, more than 1 in 10 children and 1 in 20 participants 15 years old or older are colonized with a vaccine serotype. These results will be used to evaluate the mass PCV13 campaign impact and help inform policy surrounding pneumococcal conjugate vaccine use during humanitarian crises.

## INTRODUCTION

*Streptococcus pneumoniae *(pneumococcus) is a leading cause of bacterial pneumonia, meningitis, and sepsis. Although the introduction of pneumococcal conjugate vaccines (PCVs) led to substantial declines in pneumococcal disease globally, the burden remains high in many developing countries.^1^ PCVs, recommended by the WHO for inclusion into the routine childhood immunization program for all countries using a schedule of three primary doses without a booster (3 + 0) or two primary doses with a booster (2 + 1),^2^ protect against pneumococcal carriage (a precursor to disease) and disease caused by vaccine serotypes (VTs).^3^^,^^4^ Reductions in VT carriage among vaccinated children led to declines in VT carriage and disease among unvaccinated populations (i.e., indirect effects).^5^^,^^6^ The benefits of PCVs can be eroded in settings with insecurity or war because of interruptions in vaccination programs and increased risk of pneumococcal carriage and disease owing to factors common in crisis settings, such as increased malnutrition, increased crowding, and decreased access to health care.^7^^,^^8^ Pneumococcal carriage studies can be used to monitor pneumococcal serotypes circulating in the community and changes in transmission, which may be beneficial in settings experiencing disruptions in routine PCV use as decreased coverage may lead to the re-emergence of VTs associated with invasive pneumococcal disease.

Burkina Faso is a low-income West African country located entirely in the African meningitis belt. Burkina Faso introduced a 13-valent pneumococcal conjugate vaccine (PCV13) into the routine childhood immunization program in October 2013 using a 3 + 0 schedule (at 2, 3, and 4 months), and PCV13 third-dose coverage was >90% for most years after introduction.^9^ Because of suboptimal reductions in VT carriage and disease,^10^^,^^11^ the country switched to a 2 + 1 schedule (at 2, 4, and 9 months) in June 2021 after there was emerging evidence that the 2 + 1 schedule may result in greater herd effects because of the booster dose providing a longer duration of protection among vaccinated children.^2^

In the past decade, Burkina Faso has faced increasing insecurity and a severe humanitarian crisis, resulting in over 2 million internally displaced people (IDP),^12^ health facility closures, and decreases in the availability of routine immunization services, especially in the eastern and northern parts of the country (A. Sidibe, unpublished data).^13^ The Sahel Region in northern Burkina Faso is one of the regions most afflicted by insecurity, with approximately 575,870 IDP in 2022.^14^ In 2022, the regional administrative third-dose coverage of PCV13 was 64%, a marked decline from >90% coverage between 2015 and 2018.^13^ To address this situation, from February 25 to March 2, 2022, the Ministère de la Santé et de l’Hygiène Publique conducted a mass single-dose PCV13 campaign among children ages 9–59 months old regardless of previous vaccination history in the Dori and Gorom-Gorom districts of the Sahel Region, reaching 99% (*n *= 137,308/138,015) administrative vaccination coverage.^15^

PCVs are included in the WHO Framework for Decision-Making on Vaccination in Acute Humanitarian Emergencies.^16^ However, there is limited guidance regarding the optimal number of doses or target age group for PCV use in multiage cohort (MAC) campaigns. Additionally, data on pneumococcal carriage and serotype distribution in settings experiencing declining PCV coverage, insecurity, and humanitarian crises are scarce. To address these gaps, we conducted a cross-sectional community pneumococcal carriage study approximately 1 month before the MAC PCV13 campaign. The objectives were to evaluate VT carriage immediately before the MAC PCV13 campaign and to explore factors associated with pneumococcal carriage among persons ages 1 month old or older.

## MATERIALS AND METHODS

### Study setting, design, and population.

**Figure 1.**
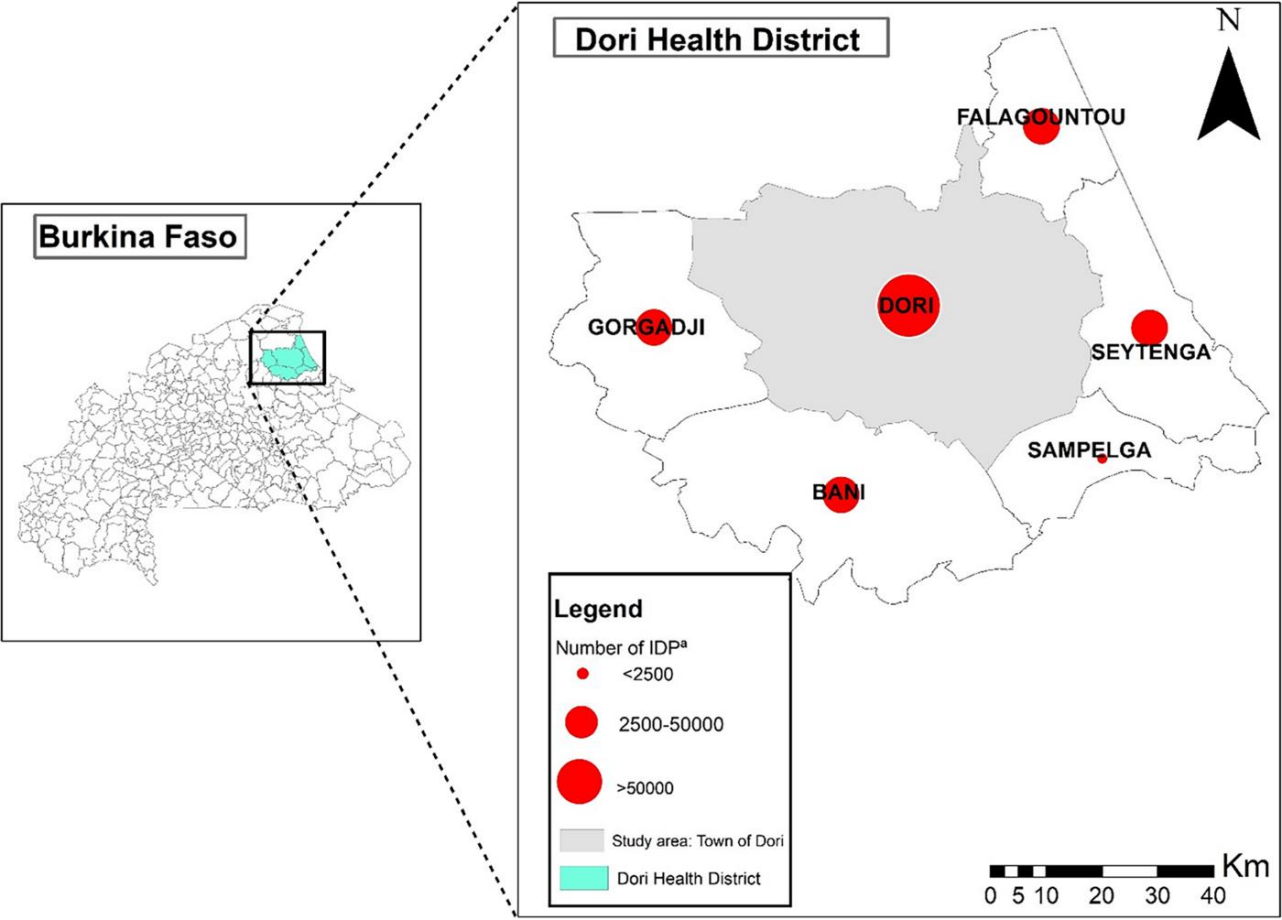
Location of Dori in the Dori Health District of the Sahel Region, Burkina Faso. IDP = internally displaced people. *The number of IDPs is as reported by the Burkina Faso National Council for Emergency Relief and Rehabilitation.^14^

In January and February 2022 before the MAC PCV13 campaign, we conducted a cross-sectional age-stratified community pneumococcal carriage study in Dori, a town in the Dori district (district population: 434,993 in 2022) and the capital of the Sahel Region of northern Burkina Faso (Figure 1).^17^ The Sahel Region is 1 of 13 administrative regions in Burkina Faso; most (88%) of the population lives in a rural setting (national: 74%),^18^ a quarter of the population faces severe food insecurity (national: 16%), and the mortality rate in children younger than 5 years old is 98 per 1,000 live births (national: 48 per 1,000 live births).^19^ We used the same recruiting methods as pneumococcal carriage studies conducted in Bobo-Dioulasso in the Hauts-Bassins Region of western Burkina Faso.^10^^,^^20^ Briefly, we used age-stratified cluster sampling to recruit participants in the community. Dori has seven sectors; in each sector, all intersections were mapped, and 30 intersections were randomly selected, with 10 backups. At each intersection, trained surveyors randomly selected a street by spinning a pen, and households were visited consecutively starting on the left. At the household, trained surveyors aimed to recruit one participant in each of five age groups: 1–11 months old, 1 year old, 2–4 years old, 5–14 years old, and 15 years old or older. In households with multiple members in the same age group, one was randomly selected. Multiple households in the same intersection were visited until one participant in each age group was enrolled for a target sample size of 1,050 participants or 210 participants per age group. Residents of Dori 1 month old or older were eligible for inclusion. IDP or refugees who were residing in Dori during participant enrollment were included. To be consistent with pneumococcal carriage studies conducted in Bobo-Dioulasso, the exclusion criteria were severe acute malnutrition or severe underlying disease reported by the participant or parent/guardian of the participant. Children with severe acute malnutrition (defined as a midupper arm circumference measurement of <115 mm) were registered at local health centers for treatment. In our assessment, surveyors probed the parent/guardian of a child to determine if the child was registered at a health center for severe acute malnutrition. If yes, the child was not eligible for inclusion.

### Data and specimen collection.

During recruitment at the household, surveyors obtained written consent from adult participants or the parent/guardian of children younger than 18 years old. After informed consent, surveyors completed a questionnaire on household characteristics and participant demographics using ODK Collect.^21^ The questionnaire was programmed to calculate age based on the date of enrollment and date of birth; if date of birth was unknown, age was self-reported in months (participants ages 1–11 months old) or years (participants ages 1 year old or older). If needed, surveyors used event calendars to help participants recall age. Vaccination history was collected for children 8 years old or younger (age eligible to receive PCV13 as infants after its introduction in 2013) in 2022. Enrolled participants received appointment cards to visit the Dori district hospital (<2 kilometers from most participant households) for collection of clinical specimens.

At the Dori district hospital, trained nurses obtained written informed consent for clinical specimen collection and completed a second questionnaire on the health history of participants. Next, trained nurses collected nasopharyngeal swabs from all participants and oropharyngeal swabs from participants 5 years old or older.^22^ Swabs were immediately placed into cryotubes containing 1 mL skim milk, tryptone, glucose, and glycerol (STGG) transport medium, and inoculated STGG was vortexed for 10–20 seconds before being placed in portable −80°C freezers until the end of sample collection.

### Laboratory methods.

When sample collection ended, the portable −80°C freezers containing the clinical specimens were transported to Centre Muraz, a national reference laboratory in Bobo-Dioulasso, and cryotubes were moved to −80°C freezers in the laboratory. For nasopharyngeal and oropharyngeal swab analysis, 200 µL of swab-inoculated STGG media were transferred to 5.0 mL Todd Hewitt broth containing 0.5% yeast extract (THY) and 1 mL rabbit serum, and they were incubated at 35–37°C for 6 hours. Cultured broth was plated on sheep blood agar and incubated in 5% CO_2_ at 35–37°C. After 18–24 hours of incubation, plates were examined for the appearance of alpha-hemolytic colonies resembling streptococci. Pneumococci were identified by susceptibility to optochin and the bile solubility test. All isolates of *S. pneumoniae* were inoculated in a preservation medium STGG and stored at −80°C. Pneumococcal serotypes were determined using the published sequential multiplex polymerase chain reaction (PCR) assay.^23^

All samples found to be negative by Centre Muraz were sent to the CDC *Streptococcus* Laboratory in Atlanta for confirmation of the negative result. Pneumococcal isolates with serotype results unresolved by multiplex PCR were further tested by Quellung reaction at the CDC; two isolates were not shipped to Atlanta, and the serotype results remained unresolved. All pneumococcal isolates determined to be nontypeable (NT) by Centre Muraz were also further tested by Quellung reaction at the CDC. Additionally, a subset (20%) of serotyped isolates was sent to the CDC for quality control of the serotype results obtained by Centre Muraz.

### Variable definitions.

VTs were defined as serotypes contained in PCV13: 1, 3, 4, 5, 6A, 6B, 7F, 9V, 14, 18C, 19A, 19F, and 23F. Two isolates that remained unresolved (i.e., not tested by Quellung reaction at the CDC) were identified as potential VTs (6A/6B/6C/6D and 7F/7A); therefore, these two isolates were categorized as VTs. All other serotypes excluding NTs were considered nonvaccine types (NVTs). Biomass fuel was defined as cooking with wood or coal only. Acute respiratory illness included runny nose, fever, and/or cough in the past 2 weeks.

## STATISTICAL ANALYSES

We conducted descriptive analyses of participants. Analyses of pneumococcal carriage prevalence were stratified by age group. For participants with both nasopharyngeal and oropharyngeal swabs collected (participants 5 years old or older), the participant was considered colonized if pneumococci were detected by culture from either sample. We described the carriage prevalence of individual serotypes using participants (all ages, children younger than 5 years old combined, and participants 5 years old and older combined) as the denominator; individuals carrying more than one serotype were counted in the numerator for each serotype. Multivariable logistic regression was used to assess factors associated with pneumococcal carriage. Covariates of etiological importance (i.e., age and recent antibiotic use) were kept in the model a priori, and other covariates with a *P*-value of <0.2 on univariable analysis were eligible for potential inclusion in the multivariable model. We used backward, forward, and stepwise automated selection to assess which variables were eligible to remain in the final adjusted model. We also explored correlation and statistical interactions between candidate variables. Data analyses were performed using SAS software v. 9.4 (SAS Inc., Cary, NC). *P*-values <0.05 were considered statistically significant.

## RESULTS

### Household and participant characteristics.

From January 14 through February 4, 2022, we recruited 1,259 participants in the community, of which 1,083 (86.0%) came to their clinic appointment and 1,079 (99.6%) consented to clinical specimen collection (Table 1). There were no socioeconomic or demographic differences among participants who came to clinic appointment and those lost to follow-up. Date of birth was known for 34.6% of participants; therefore, most participants or the parent/guardian of the participant self-reported age. Almost no households had fewer than seven members (<7: 31.7%; 7–10: 34.4%; >10: 33.9%), whereas most had four or more persons sharing a bedroom (55.1%), cooked meals with biomass fuel only (72.4%), and cooked under a semienclosed structure (60.0%). Telephones (95.0%) and radios (71.2%) were the most common household possessions among the five items that were assessed. We enrolled more female participants (59.9% of all participants combined) than males, especially among participants 15 years old or older (92.0%). Acute respiratory illness and antibiotic use in the past 2 weeks was reported by 57.7% and 5.8% of participants, respectively.

**Table 1:** Demographic and epidemiological characteristics of enrolled participants in Dori, Burkina Faso in 2022

Characteristics	*N* = 1,079	
	*n*	Percentage
Age		
1–11 months	218	20.2
1 year	216	20.0
2–4 years	217	20.1
5–14 years	215	19.9
≥15 years	213	19.7
Sex		
Female	646	59.9
Male	433	40.1
Household size		
<7	342	31.7
7–10	371	34.4
>10	366	33.9
≥4 persons sharing a bedroom		
Yes	594	55.1
No	485	44.9
Children in household attending day care or school		
Yes	678	62.8
No or do not know	401	37.2
Smoker in household		
Yes	117	10.8
No or do not know	962	89.2
Fuel type*		
Gas fuel or combination of gas and biomass fuel	298	27.6
Biomass fuel only	781	72.4
Cooking location		
Inside	96	8.9
Under semienclosed structure	647	60.0
Outside	336	31.1
Household possessions—radio^†^		
Yes	748	71.2
No	303	28.8
Household possessions—television^†^		
Yes	548	51.5
No	517	48.5
Household possessions—telephone^†^		
Yes	1,017	95.0
No	53	5.0
Household possessions—car^†^		
Yes	71	7.3
No	898	92.7
Household possessions—motorbike^†^		
Yes	710	66.3
No	361	33.7
Acute respiratory illness in past 2 weeks^†^^‡^		
Yes	622	57.7
No	456	42.3
Antibiotic use in the past 2 weeks^†^^§^		
Yes	62	5.8
No or do not know	999	94.2

* Households using wood or coal were categorized as using biomass fuel.

^†^ The denominator excludes missing data.

^‡^ Acute respiratory illness is defined as runny nose, fever, and/or cough in the past 2 weeks.

^§^ Antibiotic use in the past 2 weeks was self-reported. The participant or the parent/guardian of the participant was first asked if the participant had taken any antibiotics in the past 2 weeks. If the response was yes, then the surveyor asked for the name of the antibiotic.

### Overall and VT pneumococcal carriage prevalence.

Among all participants, overall pneumococcal carriage prevalence was 57.2% (95% CI: 54.2–60.1) (Table 2). By age group, prevalence of pneumococcal carriage was highest among children ages 1 year old (71.8%) and 1–11 months old (69.7%) and lowest among participants 15 years old or older (30.0%). Among all pneumococcal carriers, VT carriage was 22.4% (95% CI: 19.1–25.7). By age group, VT carriage among pneumococcal carriers was highest among children ages 5–14 years old (33.0%), with little variation in the other age groups (18.8% in participants 15 years old or older to 21.7% in children 1–11 months old). Among all participants, VT carriage was 12.8% (95% CI: 10.8–14.8) and ranged from 5.6% (95% CI: 2.5–8.7) in participants 15 years old or older to 17.8% (95% CI: 12.6–22.8) in children 5–14 years old.

**Table 2:** Pneumococcal carriage prevalence by age group in Dori, Burkina Faso in 2022

Prevalence	Carriage Prevalence	
	*n/N*	Percentage (95% CI)
Overall pneumococcal carriage	617/1,079	57.2 (54.2–60.1)
1–11 months	152/218	69.7 (63.6–75.8)
1 year	155/216	71.8 (65.7–77.8)
2–4 years	131/217	60.4 (53.9–66.9)
5–14 years	115/215	53.5 (46.8–60.2)
≥15 years	64/213	30.0 (23.9–36.2)
VT carriage among pneumococcal carriers*	138/617	22.4 (19.1–25.7)
1–11 months	33/152	21.7 (15.1–28.3)
1 year	30/155	19.4 (13.1–25.6)
2–4 years	25/131	19.1 (12.3–25.8)
5–14 years	38/115	33.0 (24.4–41.7)
≥15 years	12/64	18.8 (9.2–28.3)
VT carriage among all participants*	138/1,079	12.8 (10.8–14.8)
1–11 months	33/218	15.1 (10.4–19.9)
1 year	30/216	13.9 (9.3–18.5)
2–4 years	25/217	11.5 (7.3–15.8)
5–14 years	38/215	17.8 (12.6–22.8)
≥15 years	12/213	5.6 (2.5–8.7)
NVT carriage among pneumococcal carriers^†^	467/617	75.7 (72.3–79.1)
1–11 months	116/152	76.3 (69.5–83.1)
1 year	118/155	76.1 (69.4–82.9)
2–4 years	101/131	77.1 (69.9–84.3)
5–14 years	82/115	71.3 (63.0–79.6)
≥15 years	50/64	78.1 (68.0–88.3)
NVT carriage among all participants^†^	467/1,079	43.3 (40.3–46.2)
1–11 months	116/218	53.2 (46.6–59.8)
1 year	118/216	54.6 (48.0–61.3)
2–4 years	101/217	46.5 (39.9–53.2)
5–14 years	82/215	38.1 (31.6–44.6)
≥15 years	50/213	23.5 (17.8–29.2)

NVT = nonvaccine type; VT = vaccine serotype.

* VT carriage is defined as carriage with serotypes included in the 13-valent pneumococcal conjugate vaccine (1, 3, 4, 5, 6A, 6B, 7F, 9V, 14, 18C, 19A, 19F, and 23F). Two isolates that remained unresolved were identified as potential VTs (6A/6B/6C/6D and 7F/7A); therefore, these two isolates were categorized as VTs.

^†^ NVT carriage is defined as carriage with any serotype not included in the 13-valent pneumococcal conjugate vaccine, excluding NTs.

### Serotype-specific carriage prevalence.

**Figure 2.**
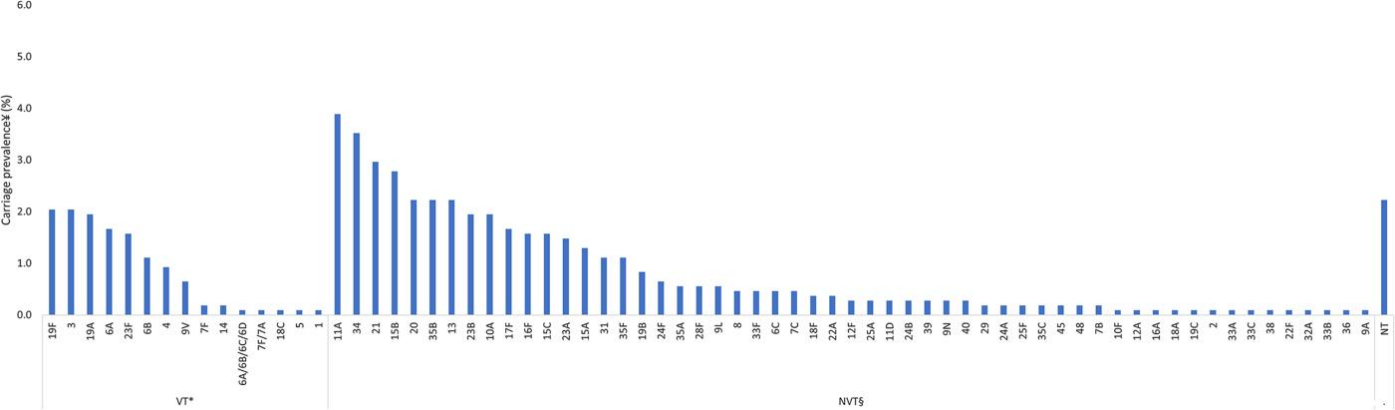
Pneumococcal carriage prevalence by serotype among all ages (*N* = 1,079) in Dori, Burkina Faso in 2022. NT = nontypeable; NVT = nonvaccine type; PCV13 = 13-valent pneumococcal conjugate vaccine; VT = vaccine serotype. *VT carriage is defined as carriage with serotypes included in PCV13 (1, 3, 4, 5, 6A, 6B, 7F, 9V, 14, 18C, 19A, 19F, and 23F). Two isolates that remained unresolved were identified as potential VTs (6A/6B/6C/6D and 7F/7A); therefore, these two isolates were categorized as VTs. ^†^NVT carriage is defined as carriage with any serotype not included in PCV13, excluding NTs. ^‡^In total, 18 participants were colonized with more than one serotype.

**Figure 3.**
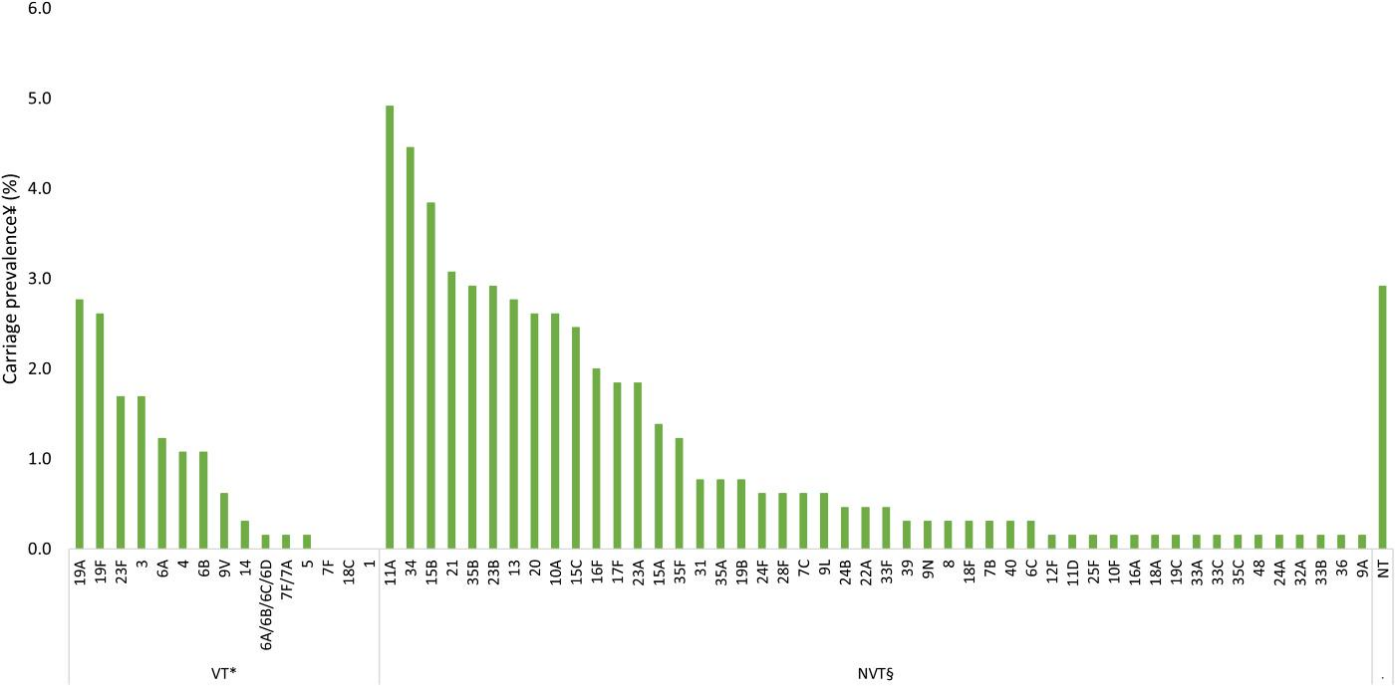
Pneumococcal carriage prevalence by serotype among children ages younger than 5 years old (*N* = 651) in Dori, Burkina Faso in 2022. NT = nontypeable; NVT = nonvaccine type; PCV13 = 13-valent pneumococcal conjugate vaccine; VT = vaccine serotype. *VT carriage is defined as carriage with serotypes included in PCV13 (1, 3, 4, 5, 6A, 6B, 7F, 9V, 14, 18C, 19A, 19F, and 23F). Two isolates that remained unresolved were identified as potential VTs (6A/6B/6C/6D and 7F/7A); therefore, these two isolates were categorized as VTs. ^†^NVT carriage is defined as carriage with any serotype not included in PCV13, excluding NTs. ^‡^In total, 5 participants were colonized with more than one serotype.

**Figure 4.**
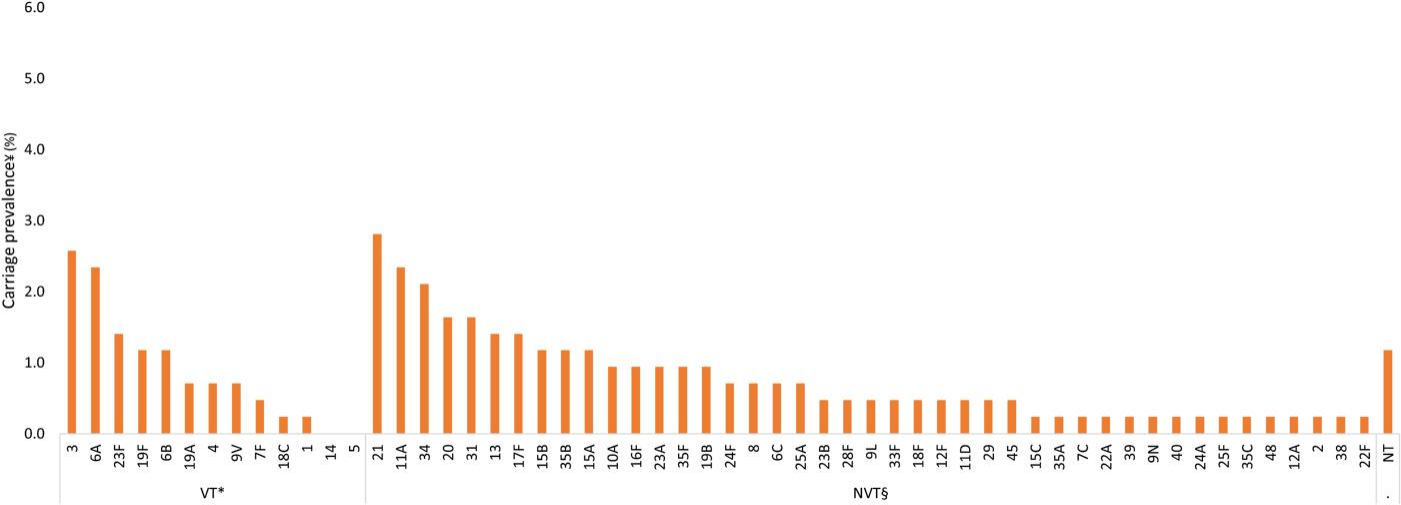
Pneumococcal carriage prevalence by serotype among participants ages 5 years old or older (*N* = 428) in Dori, Burkina Faso in 2022. NT = nontypeable; NVT = nonvaccine type; PCV13 = 13-valent pneumococcal conjugate vaccine; VT = vaccine serotype. *VT carriage is defined as carriage with serotypes included in PCV13 (1, 3, 4, 5, 6A, 6B, 7F, 9V, 14, 18C, 19A, 19F, and 23F). Two isolates that remained unresolved were identified as potential VTs (6A/6B/6C/6D and 7F/7A); therefore, these two isolates were categorized as VTs. ^†^NVT carriage is defined as carriage with any serotype not included in PCV13, excluding NTs. ^‡^In total, 13 participants were colonized with more than one serotype.

Among participants of all ages, VTs with the highest carriage prevalence were serotypes 19F (2.0%), 3 (2.0%), and 19A (1.9%), and NVTs with the highest carriage prevalence were serotypes 11A (3.9%), 34 (3.5%), and 21 (3.0%) (Figure 2). Among children younger than 5 years old, VTs with the highest carriage prevalence were 19A (2.8%), 19F (2.6%), and 23F (1.7%), and the NVTs with the highest carriage prevalence were 11A (4.9%), 34 (4.5%), and 15B (3.8%) (Figure 3). Among participants 5 years old or older, VTs with the highest carriage prevalence were 3 (2.6%), 6A (2.3%), and 23F (1.4%), and NVTs with the highest carriage prevalence were 21 (2.8%), 11A (2.3%), and 34 (2.1%) (Figure 4).

### Factors associated with pneumococcal carriage.

Participants ages 5–14 years old (adjusted odds ratio [aOR]: 0.5, 95% CI: 0.3–0.7) and 15 years old or older (aOR: 0.2, 95% CI: 0.1–0.3) were significantly less likely to be carriers of *S. pneumoniae* than children younger than 1 year old (Table 3). Participants from households cooking with biomass fuel only (aOR: 1.8, 95% CI: 1.3–2.4) were significantly more likely to be pneumococcal carriers compared with those who were not from households cooking with biomass fuel only. In addition, we observed a significant interaction between radio possession and household size. Specifically, the association between radio possession and pneumococcal carriage was only significant among households with >10 members (aOR: 2.5, 95% CI: 1.4–4.5). Cooking location and children in the household attending day care or school were associated with pneumococcal carriage on bivariate analysis; however, these variables were excluded from the multivariable model because of correlation with other variables (cooking location correlated with fuel type) and uncertainties with variable (ages of children in the household attending day care or school unknown).

**Table 3:** Factors associated with pneumococcal colonization among all ages in Dori, Burkina Faso in 2022

Characteristics	Not Colonized (*N* = 462)	Colonized (*N* = 617)	Crude OR (95% CI)	*P*-Value	Adjusted OR (95% CI)^†^
	*n* (%)*	*n* (%)*			
Age					
1–11 months	66 (14.3)	152 (24.6)	Reference	–	Reference
1 year	61 (13.2)	155 (25.1)	1.103 (0.729–1.669)	0.641	1.027 (0.670–1.575)
2–4 years	86 (18.6)	131 (21.2)	0.661 (0.445–0.984)	0.041	0.667 (0.442–1.006)
5–14 years	100 (21.6)	115 (18.6)	0.499 (0.337–0.741)	0.001	0.489 (0.325–0.736)
≥15 years	149 (32.3)	64 (10.4)	0.187 (0.124–0.281)	<0.0001	0.177 (0.116–0.272)
Sex					
Female	289 (62.6)	357 (57.9)	Reference	–	–
Male	173 (37.4)	260 (42.1)	1.217 (0.950–1.558)	0.120	–
Household size					
<7	161 (34.8)	181 (29.3)	Reference	–	–
7–10	155 (33.5)	216 (35.0)	1.240 (0.922–1.667)	0.155	–
>10	146 (31.6)	220 (35.7)	1.340 (0.995–1.806)	0.054	–
≥4 persons sharing a bedroom	264 (57.1)	330 (53.5)	0.862 (0.679–1.099)	0.232	–
Children in household attending day care or school	315 (68.2)	363 (58.8)	0.667 (0.518–0.859)	0.002	–
Smoker in household	43 (9.3)	74 (12.0)	1.328 (0.893–1.975)	0.161	–
Fuel type^‡^					
Gas fuel only or combination of gas and biomass fuel	159 (34.4)	139 (22.5)	Reference	–	Reference
Biomass fuel	303 (65.6)	478 (77.5)	1.805 (1.379–2.362)	<0.0001	1.810 (1.347–2.433)
Cooking location					
Inside	60 (13.0)	36 (5.8)	Reference	–	–
Under semienclosed structure	279 (60.4)	368 (59.6)	2.198 (1.414–3.419)	0.001	–
Outside	123 (26.6)	213 (34.5)	2.886 (1.806–4.613)	<0.0001	–
Household possessions^§^					
Radio^‖^^¶^	297 (64.3)	451 (73.1)	1.569 (1.200–2.053)	0.001	–
Among household size <7	–	–	–	–	1.227 (0.746–2.017)
Among household size 7–10	–	–	–	–	1.552 (0.984–2.447)
Among household size >10	–	–	–	–	2.511 (1.417–4.450)
Television^‖^	248 (53.7)	300 (48.6)	0.821 (0.644–1.047)	0.112	–
Telephone^‖^	438 (94.8)	579 (93.8)	0.868 (0.493–1.525)	0.622	–
Car^‖^	36 (7.8)	35 (5.7)	0.720 (0.444–1.167)	0.183	–
Motorbike^‖^	329 (71.2)	381 (61.8)	0.644 (0.496–0.836)	0.001	–
Acute respiratory illness in the past 2 weeks^‖^^#^	238 (51.5)	384 (62.2)	1.558 (1.220–1.990)	0.0004	–
Antibiotic use in the past 2 weeks^‖^**	26 (5.6)	36 (5.8)	1.055 (0.627–1.774)	0.841	–

OR = odds ratio.

* Those not colonized or colonized were used as the denominator for the percentage calculations.

^†^ Covariates of etiological importance and covariates with a *P*-value of <0.2 on univariable analysis were eligible for inclusion in the model. Adjusted OR reflects the covariates that remained in the final adjusted model using automated selection methods.

^‡^ Households using wood or coal were categorized as using biomass fuel.

^§^ The reference group for each household possession was lack of that possession.

^‖^ The denominator excludes missing data.

^¶^ Statistical interaction was found between radio and household size.

^#^ Acute respiratory illness was defined as runny nose, fever, and/or cough in the past 2 weeks.

** Antibiotic use in the past 2 weeks was self-reported. The participant or the parent/guardian of the participant was first asked if the participant had taken any antibiotics in the past 2 weeks. If the response was yes, then the surveyor asked for the name of the antibiotic.

### PCV13 vaccination history.

PCV13 history was unknown for 59.6% (*n* = 464/779) of children 8 years old or younger (Table 4). Among children 8 years old or younger with either card-confirmed or verbally reported vaccination history, 99.0% (*n* = 312/315) had no history of PCV13 receipt.

**Table 4:** Routine 13-valent pneumococcal conjugate vaccine and pentavalent vaccination history among children ages 8 years old or younger in Dori, Burkina Faso in 2022

Characteristics	*N* = 779	
	*n*	Percentage
Availability of PCV13 vaccination history		
Card confirmed or verbal report	315	40.4
Unknown	464	59.6
Receipt of PCV13 (among those card confirmed or with verbal report)	*N* = 315	
Card confirmed	1	0.3
Verbal report	2	0.6
No PCV13 receipt	312	99.0
Availability of pentavalent vaccination history		
Card confirmed or verbal report	396	50.8
Unknown	383	49.2
Receipt of pentavalent (among those card confirmed or with verbal report)	*N* = 396	
Card confirmed	69	17.4
Verbal report	247	62.4
No pentavalent receipt	80	20.2

PCV13 = 13-valent pneumococcal conjugate vaccine.

PCV13 was introduced into the routine childhood immunization program in October 2013; therefore, vaccination history is presented for children who were age eligible for PCV13 since its introduction.

## DISCUSSION

This pneumococcal carriage assessment took place in Dori, the capital of the Sahel Region, immediately before an MAC PCV13 campaign in an area with concerns of decreased PCV13 coverage and a high proportion of IDP. After 8 years of PCV13 use, more than 1 in 10 children and 1 in 20 participants 15 years old or older are colonized with a VT. This frequency of VT carriage represents a persistent threat of pneumococcal transmission, which is particularly concerning in an area with decreasing PCV13 coverage and conditions that may increase the risk for pneumococcal disease. These data will serve as a baseline for evaluating the impact of an MAC PCV13 campaign.

A pneumococcal carriage study conducted in 2020 in Bobo-Dioulasso in the Hauts-Bassins Region of western Burkina Faso found similar overall pneumococcal carriage prevalence by age group as this assessment (25.1% in participants 15 years old or older and 75.5% in children 1 year old).^10^ Compared with participants in Bobo-Dioulasso, Dori participants had risk factors that may increase pneumococcal carriage and transmission, such as greater crowding and more use of biomass fuel,^8^ making the similarity in the overall pneumococcal carriage prevalence surprising.

The VT carriage prevalence among pneumococcal carriers (Bobo-Dioulasso: 26.8%; Dori: 22.4%) and the VT carriage prevalence among all participants (Bobo-Dioulasso: 15.9%; Dori: 12.8%) were also very similar between the two assessments, despite differences in PCV13 coverage between the two regions in recent years. The Hauts-Bassins Region has been less impacted by insecurity and has maintained >90% administrative dose 3 coverage, whereas coverage in the Sahel Region was initially high but started to decline in 2019.^13^ Although prior pneumococcal carriage estimates are not available from the Sahel Region, the transmission of VT pneumococcus in the community likely declined while PCV13 coverage was high. Thus, one possibility of the comparable VT carriage in the two studies is that insufficient time had elapsed to observe re-emergence of VT circulation in Dori. Another possibility is that despite the reduction, PCV13 coverage in Dori remained sufficient to maintain indirect effects; results from a study in Lao People’s Democratic Republic found evidence of indirect effects on VT carriage in areas with moderate (maximum coverage of 60%) coverage after PCV13 introduction.^24^

Serotypes 19F, 3, and 19A were the most common VTs identified among all participants, consistent with the 2020 carriage study in Bobo-Dioulasso.^10^ Since this is the first carriage study conducted in Dori, it is unknown whether there have been changes in serotype distribution due to changes in the population in the past few years. In this study, serotype 1 was found in 0.1% of participants, which is unsurprising because serotype 1 is rarely isolated from the nasopharynx and not commonly found in pneumococcal carriage studies.^25^ Historically, serotype 1 has been the predominant disease-causing serotype in Burkina Faso, even after PCV13 introduction,^11^ and it has caused outbreaks in some West African countries.^26^^–^^28^ Despite similar VT carriage prevalence and serotype-specific carriage prevalence observed in this study compared with an area with sustained PCV13 coverage, additional monitoring of disease is warranted in areas of Burkina Faso with declining PCV13 coverage.

In 2022, administrative third-dose coverage of PCV13 was 64% in the Sahel region^13^; however, in this assessment, over half of participants 8 years old or younger had unknown PCV13 history, making PCV13 coverage in our study population largely unknown. In 2022, the Burkina Faso’s Health Emergency Response Operations Center (French acronym: CORUS) reported that 65% of health facilities in the Sahel Region had closed^29^; however, in the town of Dori, health facilities were still operating and providing routine immunization services to age-eligible children. Results from a national immunization coverage survey conducted in 2023 among children ages 12–35 months old demonstrated that PCV13 coverage in accessible areas of the Sahel Region was 86% for dose 1 and 57% for dose 3 (A. Sidibe, unpublished data). Therefore, we believe that our assessment likely underascertained PCV13 vaccination history among participants possibly because of poor card retention among IDP and among those with children who have aged out of the routine immunization program. Given the high proportion of unknown or undocumented PCV13 vaccination history in our study population, assessing the impact of the MAC PCV13 campaign on VT carriage may be challenging. Without this information, it will be unclear whether the campaign provided additional doses to previously vaccinated children or vaccinated a largely naïve population.

Consistent with previous studies,^30^ our study showed that younger age was associated with higher odds of pneumococcal carriage, whereas biomass fuel type for cooking was associated with increased odds of carriage. In addition, we observed a statistically significant interaction between radio possession and household size in relation to pneumococcal carriage. Specifically, households with radio possession and with >10 members had a 2.5-fold increase in odds of pneumococcal carriage. We believe that this interaction may be serving as a proxy for socioeconomic status among our study participants. Proxies for poverty have been well documented as factors associated with pneumococcal carriage.^30^ Although IDP status was not collected in our current study, anecdotal evidence provided by the local study staff suggests that persons who live in larger households and own radios may be more likely to be IDP and perhaps, more likely to be colonized.

This study is subject to several limitations. First, we did not capture the residence status of participants (e.g., resident, IDP, or refugee). Pneumococcal transmission in a community with a crisis-affected population may be different than that in a community with little population movement owing to changes in social contact patterns and overcrowding.^8^ Second, severe acute malnutrition was part of the exclusion criteria to be consistent with carriage surveys conducted in Bobo-Dioulasso; however, the prevalence of acute malnutrition in Dori among children ages 6–59 months old is 14%,^31^ and therefore, our results may not be representative of children in the community given that malnutrition has been associated with increased odds of pneumococcal carriage.^30^ Third, PCV13 vaccination history among children 8 years old or younger was largely unknown, perhaps because of the low card retention in an area with a large displaced population. Fourth, we believe that there were some unmeasured demographic factors, such as the residence status of participants (e.g., resident, IDP, or refugee), that may be associated with pneumococcal carriage in this assessment. Fifth, the analysis did not account for clustering of participants recruited from the same household but in different age groups. Sixth, females were overrepresented among participants 15 years old and older (92.0%), possibly because of adult men leaving Dori for extended periods of work. Lastly, the town of Dori has operating health facilities providing routine immunization services to age-eligible children and is considered more secure than surrounding areas; therefore, our results may not represent VT carriage prevalence of the Sahel Region overall.

## CONCLUSION

Despite these limitations, our study provides baseline VT carriage prevalence estimates before an MAC PCV13 catch-up campaign in the Sahel Region of Burkina Faso. Our study adds to the limited literature on pneumococcal carriage and serotype distribution in crisis-affected populations. Evidence generated from this baseline study and future studies conducted in Dori after the MAC PCV13 campaign may inform policy surrounding PCV use during humanitarian crises.
